# Enhanced Microhardness and Conductivity in a Heat-Resistant Al-Er-Zr Alloy via Optimized Thermomechanical Processing

**DOI:** 10.3390/ma19050855

**Published:** 2026-02-25

**Authors:** Chengxi Xie, Jingyang Li, Yi Lu, Shengping Wen, Shangshang Liang, Wu Wei, Xiaolan Wu, Hui Huang, Kunyuan Gao, Zuoren Nie

**Affiliations:** 1 State Key Laboratory of Materials Low-Carbon Recycling, Beijing University of Technology, Beijing 100124, China; 13648240896@163.com (C.X.); xkh@emails.bjut.edu.cn (J.L.); weiwu@bjut.edu.cn (W.W.); xiaolan.wu@bjut.edu.cn (X.W.); huanghui@bjut.edu.cn (H.H.); gaokunyuan@bjut.edu.cn (K.G.); zrnie@bjut.edu.cn (Z.N.); 2 School of Materials Science and Engineering, Taiyuan University of Science and Technology, Taiyuan 030024, China; 18810815717@163.com

**Keywords:** Al-Er-Zr alloy, thermomechanical treatment, precipitated phase, heat resistance

## Abstract

To meet the demand for high-performance heat-resistant aluminum alloy conductors in energy transmission, this study systematically explores the effects of synergistic aging and deformation treatment on the microstructure, mechanical properties, and heat resistance of Al-0.04Er-0.08Zr alloy. Through isochronous/isothermal aging, rolling with varying deformation amounts, and microstructural characterization coupled with performance testing, the following findings emerged: 425 °C represents the peak aging temperature, at which a dispersed L1_2_ structure of Al_3_(Er_1−x_Zr_x_) composite precipitates with an average size of 4 nm is formed; Dispersed L1_2_ structure Al_3_(Er_1−x_Zr_x_) composite precipitation phase achieved an alloy hardness of 49.45 HV and electrical conductivity of 58.68% IACS; the synergistic treatment of peak aging (425 °C) with 60% deformation amount yielded optimal comprehensive properties. After 150 h of isothermal annealing at 350 °C, hardness decreased by less than 5%, and the alloy demonstrated stable service life of approximately 40 years at 227 °C based on Arrhenius model extrapolation. This study reveals the synergistic regulation mechanism between deformation and aging, providing theoretical support and technical reference for developing low-cost, high thermal stability, and high-conductivity aluminum alloys.

## 1. Introduction

Against the backdrop of the global energy structure transformation and efficient development of power transmission, aluminum alloy conductors have emerged as a key material for overhead transmission lines and power transmission systems for new energy vehicles owing to their low density, excellent conductivity, and mechanical properties [1,2]. Conductors may endure prolonged exposure to operating temperatures exceeding 300 °C in high-temperature service scenarios. This requires not only maintaining high electrical conductivity to reduce transmission losses but also possessing sufficient strength and thermal stability to avoid microstructure degradation and performance decay caused by long-term high temperature exposure [3,4,5].

Al-Zr alloys serve as a typical representative of early heat-resistant aluminum alloy wires. Zr element has been observed to form Al_3_Zr nano-precipitates with L1_2_ structure with the Al matrix, which can effectively pin dislocations and grain boundaries, hinder recrystallization, and thus enhance the high-temperature stability of the alloy [6,7,8]. However, the strengthening effect of single Zr alloying is limited, and the long-term high-temperature coarsening rate of the Al_3_Zr precipitates is high, making it difficult to meet the long-term service requirements under extreme working conditions [9,10,11]. To further optimize the performance, composite microalloying has become an inevitable choice. By introducing a second alloying element to synergize with Zr, the composition, size, and distribution of the precipitates can be regulated, achieving simultaneous improvement in strengthening effect and thermal stability [12,13,14,15,16].

In the selection of microalloying elements, Sc can form a core–shell-type Al_3_(Sc,Zr) precipitates with Al and Zr, exhibiting excellent precipitation strengthening effects [17,18,19]. However, the high production cost of Sc limits its potential for large-scale application. Conversely, a rare earth element, Er, offers distinct advantages, including controllable cost and relatively abundant resource reserves. Of particular significance is the synergistic formation of the ternary composite precipitation phase Al_3_(Er_1−x_Zr_x_) in an Al matrix by Er and Zr. This phase inherits the high L1_2_ structure and exhibits enhanced ability to pin dislocations and grain boundaries. Furthermore, it significantly reduces the high-temperature coarsening rate of the precipitation phase, achieving dual benefits of precipitation strengthening and thermal stability enhancement [20,21,22].

In recent years, research on Al-Er-Zr alloys has achieved significant progress [23,24]. Existing studies have confirmed that the synergistic effect of Er and Zr can significantly enhance the alloy’s strength and thermal stability. By optimizing the aging process, the dispersed distribution of precipitated phases can be achieved, thereby improving the comprehensive performance of the alloy [25,26]. However, existing research exhibits notable limitations: most studies focus on the influence of individual process parameters on performance, lacking systematic investigation into the synergistic regulation mechanism between deformation and aging processes; the evolution patterns of precipitated phases under different treatment paths (aging followed by deformation/deformation followed by aging) and their coupling effects with performance are not clearly elucidated; and there is a lack of data on performance stability under long-term high-temperature service, making it difficult to support process optimization in practical engineering applications.

Based on the aforementioned research status, specifically the lack of systematic exploration into synergistic regulation mechanism between deformation and aging annealing, unclear evolution patterns of precipitated phases under different treatment paths and insufficient data on long-term high-temperature service stability were identified. This paper takes the Al-0.04Er-0.08Zr alloy as the research object, systematically exploring the effects of aging state and deformation treatment on the microstructure, mechanical properties, and thermal stability of the alloy. The aim is to reveal the intrinsic relationship between alloy strength, thermal stability, and microstructure. This provides theoretical support and technical reference for the development of aluminum alloys with high strengthening effect, high thermal stability, and excellent electrical conductivity.

## 2. Experimental Methods

### 2.1. Experimental Materials and Alloy Preparation

The experimental alloy was prepared using the ingot metallurgy method. The smelting raw material was high-purity aluminum (99.99%), and the microalloying elements Er and Zr were added in the form of master alloys (Al-6.0 wt.%Er, Al-4.0 wt.%Zr). The smelting process was carried out in a SG2-7.5-12-type crucible resistance furnace using a graphite clay crucible. The high-purity aluminum was first melted at 780~800 °C, and then the Al-Er and Al-Zr master alloys were added according to the alloy composition design. After all the raw materials were melted, an appropriate amount of C_2_Cl_6_ was added for degassing, followed by secondary refining, standing, and slagging. The casting temperature was 710~730 °C.

The actual composition of the experimental alloy was determined using the ICP-AES method with a LEEMANSPEC-E (PerkinElmer, Shelton, CT, USA) inductively coupled plasma atomic emission spectrometer. Table 1 compares the designed composition and the actual composition of the experimental alloy. The actual composition is close to the designed composition. For ease of description, the alloy is referred to as the designed composition throughout this article.

### 2.2. Aging Treatment of Alloys

In order to facilitate the better solid solution of Er and Zr elements in the alloy into the aluminum alloy matrix, the alloy samples after melting and casting were all subjected to solid solution treatment at 640 °C for 20 h. There are mainly two methods for aging treatment: one is isochronous aging—the temperature is continuously raised from 150 °C to 600 °C with a temperature rise step of 25 °C, the sample is held at each temperature for 3 h [25,27], and then cooled to room temperature by water; the second is isothermal aging—aging at a constant temperature of 350 °C.

### 2.3. Deformation Treatment of Alloys

#### 2.3.1. Alloy Aging Treatment Followed by Deformation Processing

To explore the effect of deformation treatment on the precipitation process of precipitated phases during subsequent aging treatment of alloys, cold rolling deformation followed by aging treatment was adopted. Firstly, the solid solution-treated alloy was subjected to cold rolling (cold rolling deformation was performed using a double-roll reversible cold rolling mill, with a reduction of 2 mm per pass.), with deformation amounts of 0%, 20%, 60%, and 90%, respectively. The deformation amount is calculated through thickness reduction rate, and the formula is: deformation amount (%) = (original thickness − thickness after rolling)/original thickness × 100%. The original thickness of the sample is 30 mm, and it is rolled to the target thickness through multiple passes (24 mm after 20% deformation, 12 mm after 60% deformation, and 3 mm after 90% deformation). Subsequently, the deformed samples were subjected to isochronal aging and isothermal aging, respectively.

#### 2.3.2. Alloy Pre-Deformation and Aging Treatment

To explore the impact of precipitation on the deformation microstructure of alloys in different aging states (under-aged, peak-aged, and over-aged) after rolling deformation with the same amount of deformation, alloys with different aging states were obtained through the pre-aging treatment. Subsequently, these alloys were subjected to cold rolling deformation with a deformation amount of 60%. Afterwards, the samples after aging and deformation were treated with recrystallization annealing. The recrystallization annealing temperature ranged from 150 °C to 600 °C, with a step length of 25 °C/1 h. The dimensions of alloy specimens at different stages are as follows: after solution treatment, aluminum ingots are processed into plates measuring 30 mm thickness, 50 mm width, and 100 mm length. Specimens for microstructure observation and property testing were cut to the following specifications: 10 mm × 10 mm × 6 mm (for microhardness and electrical conductivity testing) and 3 mm × 3 mm × 0.1 mm (for TEM observation).

### 2.4. Microstructure Observation and Performance Testing

Using the HXD-1000 microhardness tester (Shanghai Cai Kang Optical Instrument Factory, Shanghai, China), the microhardness of alloy samples was tested and analyzed. The load size was set at 200 gf, and the loading time was selected as 10 s. Each sample was measured for microhardness at least 10 times, and the average value of the 10 measurements was taken as its microhardness. The conductivity was measured using the German HOSFERSTER conductivity meter SIGMATEST2.069 (German HOSFERSTER, Reutlingen, Germany), with the measurement unit being %IACS. The measurement temperature was at 25 °C, and the measurement frequency was selected as 60 kHz. Each sample was measured six times and the average value was taken.

The microstructure was observed by providing Z-contrast images through transmission electron microscope (TEM, JEOL, Tokyo, Japan, JEM2010). A double-jet electrochemical device was used to prepare thin disks with a diameter of 3 mm. The double-jet device operated in a solution composed of 25% HNO_3_ and 75% CH_3_OH at a temperature of −20 °C and a voltage of approximately 12–15 V. The TEM observation was carried out using a transmission electron microscope on a Cs-corrected Titan-G2 (FEI Company, Hillsboro, OR, USA), which operated at an accelerating voltage of approximately 300 kV with a probe convergence half-angle of 21.4 mrad and a collection half-angle of 53 to 200 mrad. Before the TEM observations, all the prepared samples underwent 8 min of plasma cleaning. The mean circular-equivalent radii of the nano-precipitates were determined utilizing Image-Pro Plus 6.0 software and manually traced areas (at least 100 measurements per micrograph) on TEM micrographs.

## 3. Results and Discussion

### 3.1. Isothermal Aging Behavior of Al-Er-Zr Alloys

**Figure 1:**
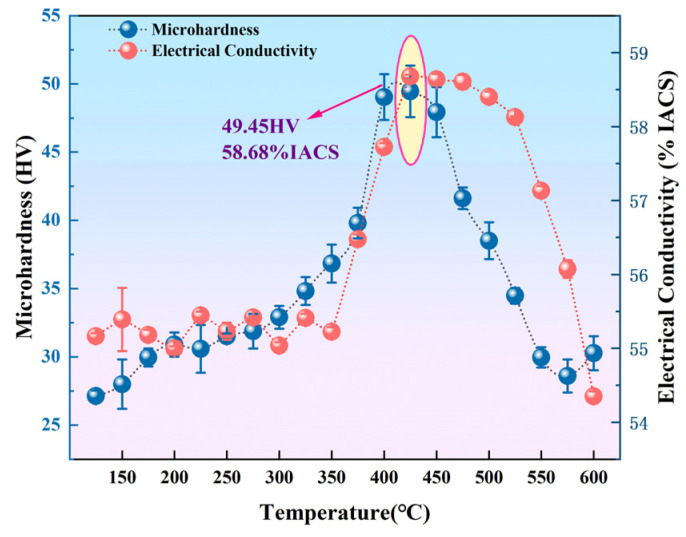
Temperature–hardness–conductivity relationship curves for Al-0.04Er-0.08Zr alloy after solution treatment followed by isochronal aging (3 h per temperature).

Figure 1 shows the temperature–hardness–electrical conductivity relationship curves for the isothermal aging of Al-0.04Er-0.08Zr alloy after solution treatment. Microhardness continuously increases with rising temperature between 150 °C and 425 °C, peaking at 49.45 HV at 425 °C. Beyond 425 °C, microhardness decreases significantly with temperature, dropping to approximately 30 HV at 600 °C. Electrical conductivity generally increases with temperature between 150 °C and 450 °C, peaking at 425 °C (58.68% IACS). Above 425 °C, conductivity gradually decreases with increasing temperature, dropping to 54.35% IACS at 600 °C.

**Figure 2:**
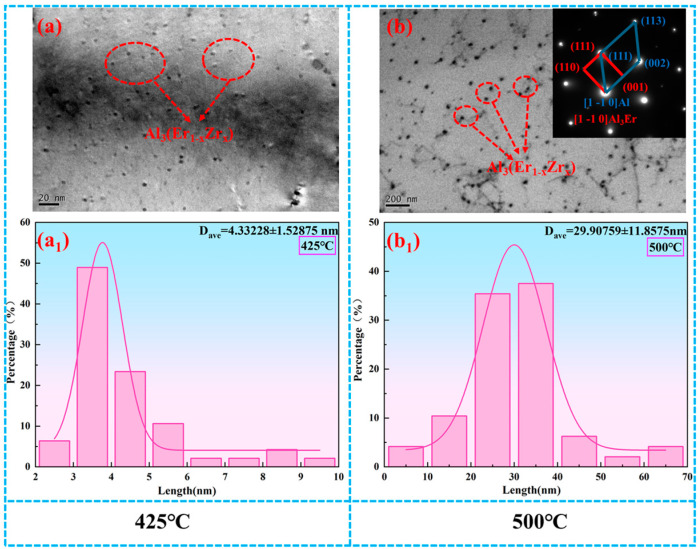
Bright-field TEM micrographs taken along the [011] Al zone axis. (**a**,**a_1_**) TEM micrograph of Al-0.04Er-0.08Zr alloy isochronal aged at 425 °C, (**b**,**b_1_**) TEM micrograph of Al-0.04Er-0.08Zr alloy isochronal aged at 500 °C, along with corresponding SAED pattern of Al_3_(Er_1−x_Zr_x_) phase.

Figure 2 TEM micrographs of Al-0.04Er-0.08Zr at peak age hardening (425 °C) and over-aged state (500 °C). As shown in Figure 2a, precipitates with an average size of approximately 4 nm and a dispersed distribution are observed at peak aging. Based on previous research by our group, these particles are identified as the ternary compound phase Al_3_(Er_1−x_Zr_x_) with an L1_2_ structure [28,29]. As shown in Figure 1, at 425 °C, the dispersed, fine Al_3_(Er_1−x_Zr_x_) precipitates (4 nm) exceed the critical size for shear separation of precipitates in aluminum-based alloys, thereby preventing dislocations from shearing through the precipitates. Instead, dislocations form annular chains around the precipitates via the Orowan bypass mechanism, impeding dislocation motion. Simultaneously, the precipitation process consumes solute elements, erbium and zirconium, reducing lattice distortion and electron scattering. This mechanism underpins both the maximum hardness (49.45 HV) and the maximum electrical conductivity (58.68% IACS).

At the over-aged state (500 °C), the precipitation phase in the Al matrix undergoes significant coarsening, reaching a size of approximately 30 nm, yet remains uniformly distributed, as shown in Figure 2b. At this stage, the alloy exhibits a significant decrease in microhardness and a slight reduction in electrical conductivity. The weakened lattice coherence between coarse-grained precipitates and the aluminum matrix diminishes the coherent strain strengthening effect. Simultaneously, the reduced number of precipitates and increased inter-precipitate spacing diminish Orowan slip resistance, as fewer particles impede dislocation slip. This dual effect of Orowan strengthening failure and coherent strain reduction leads to a sharp microhardness decline, consistent with behavior reported in other Al-L1_2_ precipitation systems [29,30,31]. The slight decrease in electrical conductivity reflects the essentially constant volume fraction of the precipitates despite limited solute dissolution in the coarsened precipitates slightly increasing electron scattering. At 600 °C, further dissolution of the precipitates increases the solute elements in the matrix, leading to a dual decrease in both microhardness and electrical conductivity.

### 3.2. The Effect of Deformation Treatment on the Aging Behavior and Microstructure of Alloys

#### 3.2.1. Effect of Rolling Treatment on Subsequent Aging Microhardness and Electrical Conductivity of Al-0.04Er-0.08Zr Alloy

**Figure 3:**
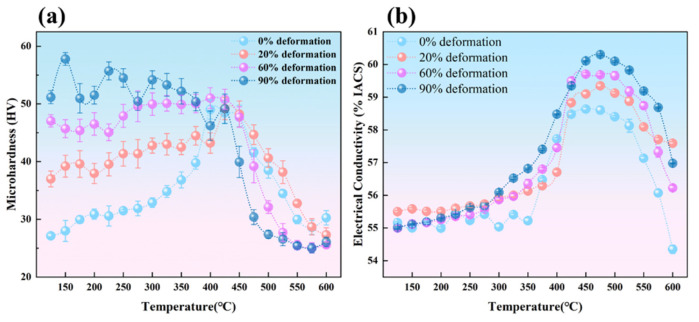
Microhardness (**a**) and electrical conductivity (**b**) variation curves of Al-0.04Er-0.08Zr alloy during isochronal aging (3 h per temperature) after deformation.

Figure 3 shows the temperature-dependent curves of isothermal aging microhardness versus electrical conductivity for Al-0.04Er-0.08Zr alloy in the solution-treated state after rolling with different deformation amounts. As shown in Figure 3a, the strength of the rolled alloy increases significantly with increasing deformation due to work hardening. The microhardness of the 90% deformation alloy exceeds 50 HV, doubling that of the solution-treated alloy. However, for subsequent aging treatment, the effect of deformation on enhancing the peak aging microhardness is not pronounced. Except for the alloy treated with 90% deformation, the peak aging microhardness values for the other deformation levels are similar, all reaching a microhardness peak at 425 °C. Among these, the microhardness of the undeformed and 20% deformation-treated alloys is approximately 48 HV. The alloy with 60% deformation exhibited a higher peak microhardness of 51.00 HV. For the 90% deformation alloy, microhardness increased at lower temperatures (150–250 °C) as aging progresses. As temperature further increased (above 250 °C), the microhardness curve exhibited a decreasing trend. At 425 °C, microhardness dropped to 48.00 HV, lower than that of the 60% deformation alloy. This indicates that for the 90% deformation alloy, the prior deformation strengthening effect was significant, but subsequent aging treatment did not lead to a further strength enhancement.

In summary, strain hardening does not significantly enhance subsequent aging strengthening, and no simple additive relationship exists between strain hardening and aging strengthening. This is partly because the resistance to dislocation motion imposed by precipitation phases and strain-induced microstructures are not independent processes; they exhibit mutual coupling and shielding effects. That is, if dislocation motion is hindered by one factor, the other factor cannot exert its full effect. On the other hand, during aging, the deformed microstructure undergoes recovery phenomena, which also contributes to the weakening of the work hardening effect.

Figure 3b shows the temperature-dependent electrical conductivity curves of Al-0.04Er-0.08Zr solid solution alloys after rolling with different deformation amounts. The electrical conductivity of the rolled state with varying deformation amounts is close to that of the solid solution alloy without deformation, further indicating that deformation treatment has a minor effect on the alloy’s electrical conductivity. The temperature-dependent conductivity trends for alloys with different deformation levels are fundamentally consistent: conductivity increases with rising aging temperature, peaks around 475 °C, and then decreases with further temperature elevation. However, the overall conductivity curve exhibits a certain degree of enhancement as deformation increases. The greater the pre-deformation amount, the higher the peak conductivity during aging. The conductivity peak of the 90% deformation alloy reached 60.50% IACS, representing an approximate 2.0% IACS increase compared to the peak conductivity of the non-deformed alloy. Moreover, as deformation increases, the temperature at which conductivity begins to rise decreases. That is, at the same temperature, alloys with greater deformation generally exhibit higher conductivity. This occurs because deformation increases the alloy’s dislocation density, forming pathways for rapid atomic diffusion. This promotes the precipitation and growth of secondary phases. Since conductivity is primarily influenced by solid solution atoms, increased deformation leads to both a faster rate of conductivity increase and a higher peak conductivity value.

#### 3.2.2. Microstructural Observation

**Figure 4:**
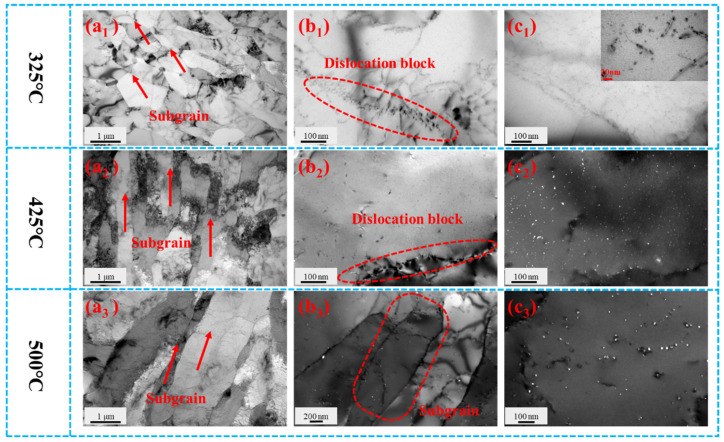
BF TEM micrographs of Al-0.04Er-0.08Zr solid solution with 60% deformation after rolling aged at isochronal temperatures of 325 °C (**a_1_**–**c_1_**), 425 °C (**a_2_**–**c_2_**), and 500 °C (**a_3_**–**c_3_**).

**Figure 5:**
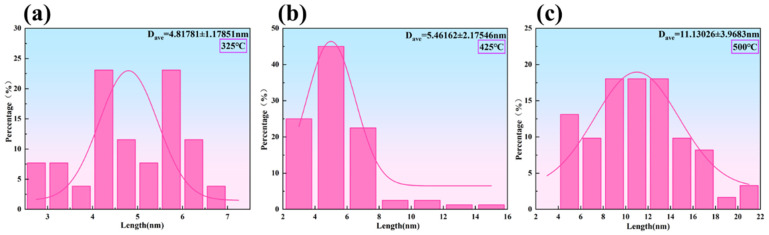
Grain diameter of the precipitated phase at different temperatures: (**a**) 325 °C, (**b**) 425 °C, and (**c**) 500 °C.

Figure 4(a_1_–c_1_) show TEM microstructures of the Al-0.04Er-0.08Zr alloy after solution treatment, 60% deformation rolling, and isothermal aging at 325 °C. Low-magnification images reveal distinct subgrains, indicating no recrystallization occurred at this temperature. For aluminum alloys without precipitates, recrystallization typically occurs during annealing at 325 °C. Since isothermal aging involves 3 h holds at each temperature—more stringent than recrystallization annealing conditions—precipitates must form to inhibit recrystallization. The aging microhardness curve in Figure 3a shows a significant increase in microhardness at this temperature compared to the initial aging stage, further supporting the formation of precipitates. However, no distinct precipitates were observed at lower magnifications in Figure 4(a_1_,b_1_). Only at higher magnifications were a few nanoscale precipitates identified, with sizes around 5 nm (Figure 5a).

The microstructure of the Al-0.04Er-0.08Zr alloy after 60% deformation rolling and isothermal aging at 425 °C is shown in Figure 4(a_2_–c_2_). The low-magnification SEM image in Figure 4(a_2_) reveals distinct subgrains, indicating that no recrystallization occurred at this temperature. The higher magnification TEM images in Figure 4(b_2_–c_2_) reveal pronounced dislocation pileups and abundant precipitates with sizes around 5.5 nm (Figure 5b). These precipitates are generally uniformly distributed and preferentially form along dislocation lines and subgrain boundaries. They effectively impede the motion of dislocations and subgrain boundaries, thereby inhibiting recrystallization. The peak aging temperature corresponds to 425 °C. Compared to the alloy subjected to direct aging treatment from the solution-treated state (Figure 2a, where the peak-aged precipitation phase size is approximately 4 nm), the precipitation phase size is slightly larger.

Figure 4(a_3_–c_3_) shows the microstructures of the Al-0.04Er-0.08Zr alloy after 60% deformation rolling followed by isothermal aging at 500 °C. At low magnification, subgrains formed from the rolled structure after annealing are still visible, indicating incomplete recrystallization. This evolution resembles the recrystallization annealing microstructure of the aging + deformation-treated alloy depicted in Figure 7e,f. Although the hardness curve suggests recrystallization should have been completed, the microstructural features indicate otherwise. Figure 4(b_3_) reveals numerous second-phase particles, some of which exist at subgrain boundaries. These particles impede subgrain boundary motion, thereby delaying the onset of recrystallization. Compared to recrystallization annealing, the isothermal aging conditions were more stringent, with a 3 h soak at each temperature point. Moreover, samples at each temperature had previously undergone all preceding heat treatments. Yet, under these conditions, the precipitated phases still effectively hindered recrystallization, demonstrating the excellent thermal stability of the deformed microstructure in the Al-0.04Er-0.08Zr alloy. At higher magnifications (Figure 4(c_3_)), numerous precipitates with diameters around 10 nm are visible (Figure 5c), exhibiting significant coarsening compared to the peak-aged state. This coarsening of precipitates is likely the primary factor contributing to the sharp decrease in hardness.

### 3.3. Effect of Aging Condition on Subsequent Rolling Properties and Microstructure of Al-Er-Zr Alloys

#### 3.3.1. Effect of Aging Condition on Subsequent Rolling Strength and Electrical Conductivity of Al-Er-Zr Alloys

**Figure 6:**
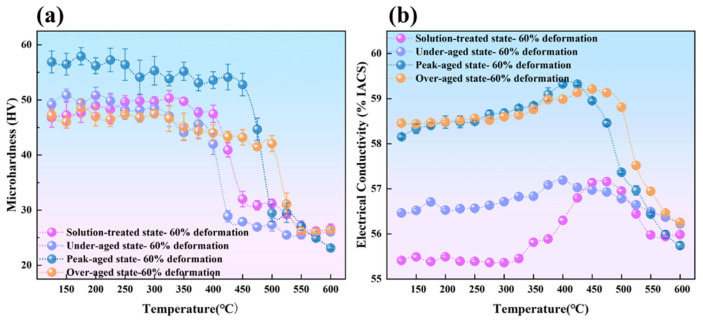
Recrystallization annealing microhardness (**a**) and electrical conductivity (**b**) variation curves for Al-0.04Er-0.08Zr alloy in different aging states after 60% deformation rolling treatment.

Based on the isochronal aging curves shown in Figure 1, different aging conditions for the Al-Er-Zr alloy were determined. Three alloys with distinct aging states were obtained: under-aged (375 °C), peak-aged (425 °C), and over-aged (500 °C). Figure 6a shows the microhardness versus annealing temperature curve for Al-0.04Er-0.08Zr alloy in different aging states after 60% deformation rolling and subsequent recrystallization annealing. In the rolled condition, the peak-aged alloy exhibits the highest hardness, reaching approximately 57 HV. This indicates that under peak aging conditions, the precipitation phases exert the greatest hindrance and enhancement on dislocation motion, resulting in the highest work hardening capability. In the over-aged state, although individual precipitation phases are larger and provide stronger dislocation hindrance, their number density is lower than that in the peak-aged state, leading to a work hardening capability inferior to that of the peak-aged condition. The solution-treated alloy contains no precipitates, yet solution atoms contribute to work hardening, resulting in microhardness values close to those of the under-aged and over-aged states. Using the sudden drop in microhardness as the recrystallization onset temperature, the over-aged Al-0.04Er-0.08Zr alloy exhibits the highest recrystallization temperature (525 °C) after 60% deformation. The peak-aged Al-0.04Er-0.08Zr alloy exhibits a recrystallization temperature of 475 °C after the same deformation treatment; next is the solution-treated alloy at 425 °C after 60% deformation; and the under-aged state shows the relatively lowest temperature, around 400 °C. The recrystallization onset temperatures decrease in the following order: over-aged > peak-aged > under-aged. In contrast, the solution-treated alloy contained no precipitates, and its rolled microstructure exhibited high stability. Its recrystallization onset temperature reached 425 °C, significantly higher than typical aluminum alloys (generally around 275–325 °C). This may be attributed to the formation of precipitates during recrystallization annealing in the presence of deformation dislocations, which hindered recrystallization.

Figure 6b shows the variation curves of electrical conductivity with recrystallization annealing treatment for Al-0.04Er-0.08Zr alloy in different aging conditions after 60% deformation rolling. In the rolled condition, the peak-aged and over-aged states exhibit higher conductivity levels at 58.16% IACS and 58.46% IACS, respectively, with the over-aged state slightly exceeding the peak-aged state. The under-aged state shows lower conductivity at 56.40% IACS, while the solution-treated state exhibits the lowest conductivity at 55.30% IACS. Under identical deformation conditions, the primary factor influencing conductivity is the state of solute atoms. Solution-dissolved atoms exert the greatest effect on conductivity, which subsequently increases upon precipitation phase formation. Consequently, conductivity follows the pattern: over-aged > peak-aged > under-aged > solution-treated.

As the annealing temperature increases, electrical conductivity shows a certain degree of improvement. Among these, the solid solution state exhibits the most significant increase in conductivity, with a peak conductivity around 57.20% IACS, representing an improvement of approximately 2.0% IACS compared to the rolled state. Even the peak-aged and over-aged alloys exhibited conductivity peaks around 400 °C. This confirms precipitation phase formation during recrystallization annealing of the solution-treated alloy. The conductivity curve shows an increase starting at 325 °C, indicating precipitation initiation at this temperature. Thus, the solution-treated alloy demonstrates high thermal stability during recrystallization annealing. The increase in conductivity for peak-aged and over-aged alloys indicates that deformation-induced dislocations promote further precipitation of solute atoms within the alloy. After reaching the peak temperature, further temperature increases cause conductivity to decline. This is due to the dissolution of precipitates at elevated temperatures. The dissolved atoms increase electron scattering within the aluminum matrix, thereby reducing conductivity.

#### 3.3.2. Effect of Aging Condition on the Microstructure of Alloys for Subsequent Rolling

**Figure 7:**
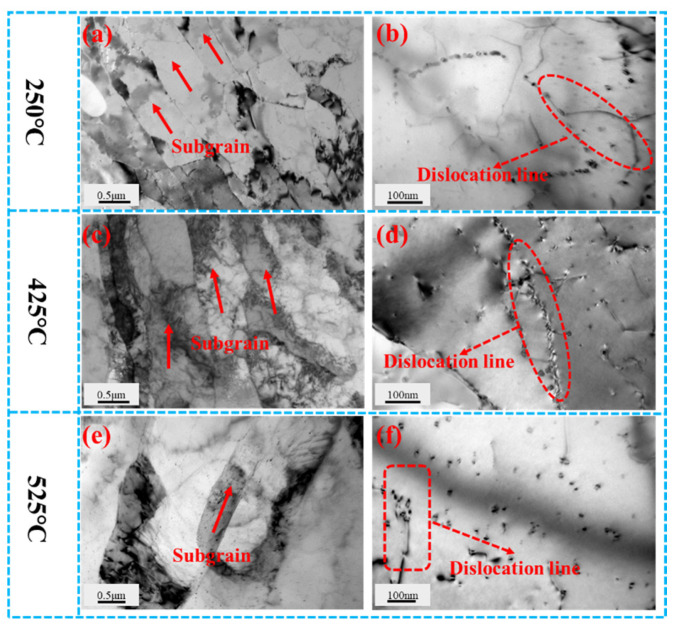
BF TEM micrographs of Al-0.04Er-0.08Zr over-aged alloy after 60% deformation annealed at 250 °C/1 h (**a**,**b**), 425 °C/1 h (**c**,**d**), and 525 °C/1 h (**e**,**f**).

Figure 7a,b show TEM microstructural images of the over-aged Al-0.04Er-0.08Zr alloy after 60% deformation and 1 h annealing at 250 °C. At low magnification, distinct deformation microstructure is visible, with dislocations randomly distributed within subgrains. At high magnification, dislocation lines pinched by secondary phases are observable, indicating that secondary phases impede dislocation motion.

Figure 7c,d shows TEM microstructural images of the aged Al-0.04Er-0.08Zr alloy after 60% deformation and 1 h of annealing at 425 °C. Rolling texture remains visible at low magnification, with most dislocations migrated to subgrain boundaries. High magnification also reveals secondary phase pinning of dislocations. The microhardness versus recrystallization temperature curve in Figure 6a indicates that microhardness has not yet begun to decrease at this temperature. The microstructure shown in Figure 7c,d confirms that recrystallization has not occurred at 425 °C due to the hindrance of dislocation motion by precipitates and subgrain boundaries.

Figure 7e,f present TEM microstructures of the over-aged Al-0.04Er-0.08Zr alloy after 60% deformation followed by 1 h annealing at 525 °C. Low magnification still reveals that the subgrained structure formed from the rolled microstructure after annealing, with most dislocations migrated to the subgrain boundaries. High magnification also reveals numerous uniformly distributed precipitates and their hindrance to dislocation movement. The microhardness curve in Figure 6a shows a significant decrease in alloy microhardness at this temperature, generally indicating completed recrystallization. However, the microstructure indicates that recrystallization is incomplete. This demonstrates that even at the elevated temperature of 525 °C, the precipitation still exerts sufficient hindrance on subgrain boundary motion to inhibit the formation and growth of recrystallized grains. Furthermore, although the alloy did not recrystallize at such a high temperature, the recovery process was already quite thorough. Consequently, the driving force for recrystallization was actually quite low, making it difficult for recrystallization to occur. The decrease in microhardness observed on the microhardness curve is likely due to the coarsening of the precipitated phase. As shown in the aging curve in Figure 1, the precipitated phase rapidly coarsened at 525 °C, causing a rapid decrease in hardness.

### 3.4. Heat Resistance of Alloys

Heat-resistant aluminum alloy wires need to withstand high temperatures for extended periods during use, thus the long-term stability of their properties at a certain temperature must be considered. The Al-0.04Er-0.08Zr alloy, which underwent peak aging and 60% deformation through rolling, and the Al-0.04Er-0.08Zr alloy, which underwent solution treatment followed by 60% deformation through rolling, were then subjected to isothermal annealing at 350 °C to investigate the changes in microhardness with annealing time.

**Figure 8:**
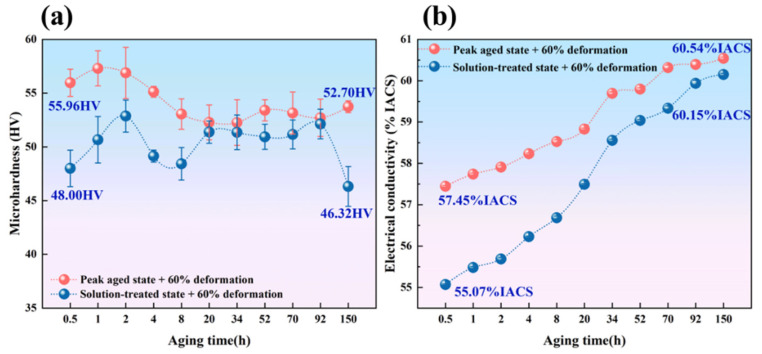
Microhardness (**a**) and electrical conductivity (**b**) variation curves of 60.54 alloy in the Peak aging condition after 60% deformation rolling treatment during isothermal annealing at 350 °C.

The measured curve of microhardness variation with annealing time is shown in Figure 8a. The microhardness of the alloys with two different treatments did not show significant changes during the experiment and remained at a high level. After aging for 150 h, the decrease in microhardness values was no more than 5%. The peak microhardness of the alloy after peak aging was 55.96 HV, while the peak microhardness after isothermal aging was 57.30 HV (1 h), with a minimum value of 52.70 HV, representing a decrease of 3.98%. This indicates that the precipitation phase formed during aging treatment can remain stable for extended periods at 350 °C, effectively inhibiting dislocation and subgrain boundary motion, thereby significantly enhancing the alloy’s thermal stability. The microhardness of the solution-treated and deformed alloy exhibited a trend of initial increase, followed by decrease, then recovery, and finally decline. The microhardness of the solution-treated and deformed alloy was 48 HV. After isothermal aging, the peak microhardness reached 52.86 HV (2 h) and finally decreased to 46.32 HV after 150 h, representing a decrease of 3.5%.

Figure 8b shows the evolution of electrical conductivity versus annealing time during isothermal annealing at 350 °C for two different treatment methods of the Al-0.04Er-0.08Zr alloy. The figure indicates that conductivity consistently increases throughout the annealing process. After 150 h of isothermal aging, the conductivity of the peak-aged alloy increased from 57.45% IACS to 60.54% IACS, representing an approximately 3% IACS increase compared to the pre-annealed state. For the solution-treated and deformed alloy, conductivity rose from 55.07% IACS to 60.15% IACS, showing a 5% IACS increase relative to the pre-annealed value. Deformation promotes further precipitation of solution-treated atoms.

In summary, the “peak-aged state + 60% deformation” alloy exhibits superior combined performance in terms of hardness and electrical conductivity. Using heat resistance tests combined with Arrhenius curves [32], the alloy’s long-term operating temperature can be inferred.

**Figure 9:**
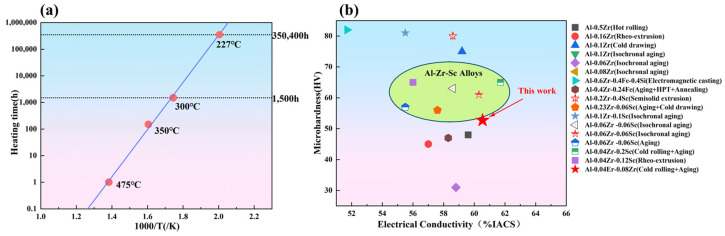
(**a**) Heat resistance Arrhenius curve of Al-0.04Er-0.08Zr alloy; (**b**) Microhardness—electrical conductivity correlation map of aluminum-based alloys [14,33,34,35,36,37,38,39,40,41,42,43,44].

In this work, the “peak-aged state + 60% deformation” alloy(Al-Er-Zr alloy) achieved a microhardness of 52.7 HV after annealing. High-temperature aging tests (Figure 9a) showed that at 475 °C for 1 h, 350 °C for 150 h and 300 °C for 1500 h, the microhardness retention ratio remained above 90%. Fitting the data using the Arrhenius equation revealed that the alloy has a relatively short service life at 475 °C. However, when the temperature is reduced to 227 °C, the service life extends to 350,000 h (approximately 40 years), with the strength retention ratio still reaching 90%. This indicates that 1# alloy optimized by annealing has excellent thermal stability and can be used in environments of up to 227 °C for long-term service.

Combining with the aluminum alloy microhardness–electrical conductivity correlation map in Figure 9b, Al-Zr-Sc alloys (light green area) exhibit better electrical conductivity and microhardness. In contrast, the Al-Er-Zr alloy in this study (labeled “this work”) exhibits slightly lower microhardness than the Al-Zr-Sc alloy, but its electrical conductivity falls within the same range. Moreover, the cost of Al-Er-Zr alloy is only 1/80 of that of Al-Zr-Sc alloy, which makes it more cost-effective. Combined with its excellent thermal performance, this alloy demonstrates significant advantages in both microhardness–conductivity compatibility and long-term high-temperature reliability, offering a new direction for the performance optimization of aluminum-based conductor materials.

## 4. Conclusions

We systematically explored the effects of aging and deformation treatment on the microstructure, mechanical properties, and thermal stability of the Al-0.04Er-0.08Zr alloy. The following conclusions are drawn:

After solution treatment at 640 °C for 20 h, 425 °C was identified as the peak aging temperature. At this temperature, a diffusely distributed L1_2_ structure Al_3_(Er_1−x_Zr_x_) ternary composite precipitation phase formed with an average size of approximately 4 nm. Dispersed L1_2_ structure Al_3_(Er_1−x_Zr_x_) ternary composite precipitation phase also formed. This phase effectively pinned dislocations and subgrains, achieving an alloy hardness of 49.45 HV and electrical conductivity of 58.68% IACS, serving as the key to alloy strengthening and enhanced electrical conductivity.

In the synergistic treatment of deformation and aging, the “peak aging + 60% deformation” process achieves optimal comprehensive properties: after 150 h of isothermal annealing at 350 °C, alloy hardness decreased by less than 5%, while electrical conductivity increased to 60.54% IACS. The analysis of the Arrhenius curve indicates the alloy can stably operate at 227 °C for approximately 40 years with 90% strength retention, demonstrating outstanding long-term high-temperature stability.

Compared to high-cost Al-Zr-Sc alloys, the low-cost Al-Er-Zr alloy developed in this study achieves comparable electrical conductivity while exhibiting superior thermal stability. This provides a viable pathway for low-cost, large-scale application of heat-resistant aluminum alloy conductors.

## Figures and Tables

**Table 1:** Composition of experimental alloys (at%).

Experimental Alloy	Design Composition (at%)		Actual Composition (at%)	
	Er	Zr	Er	Zr
Al-0.04Er-0.08Zr	0.04	0.08	0.037	0.085

## Data Availability

The original contributions presented in this study are included in the article. Further inquiries can be directed to the corresponding authors.

## References

[1] Parvizi P., Jalilian M., Mirazizi P.S., Zangeneh M.R., Amidi A.M. (2025). Mechanical and physical properties of aluminum and its alloys for electrical conductors: A review. Next Mater..

[2] Czerwinski F. (2024). Aluminum alloys for electrical engineering: A review. J. Mater. Sci..

[3] Lunn K.F., Apelian D. (2025). Thermal and Electrical Conductivity of Aluminum Alloys: Fundamentals, structure-property relationships, and pathways to enhance conductivity. Mater. Sci. Eng. A.

[4] Zhang J., Peng J. (2023). A review on aluminum alloy conductors influenced by alloying elements and thermomechanical treatments: Microstructure and properties. J. Mater. Res..

[5] Dong H.-R., Li X.-Q., Li Y., Wang Y.-H., Wang H.-B., Peng X.-Y., Li D.-S. (2022). A review of electrically assisted heat treatment and forming of aluminum alloy sheet. Int. J. Adv. Manuf. Technol..

[6] Wu S., Yin J., Luo Y., Huang J., Wang Y., Liu Y., Zhong Y. (2023). A high strength Al-Zr alloy with good electrical conductivity by combining gas-assisted continuous casting and extrusion with subsequent thermomechanical treatment. Mater. Today Commun..

[7] Srinivasarao B., Suryanarayana C., Oh-Ishi K., Hono K. (2009). Microstructure and mechanical properties of Al–Zr nanocomposite materials. Mater. Sci. Eng. A.

[8] Tamim R., Mahdouk K. (2018). Thermodynamic reassessment of the Al–Zr binary system. J. Therm. Anal. Calorim..

[9] Souza P.H.L., de Oliveira C.A.S., do Vale Quaresma J.M. (2018). Precipitation hardening in dilute Al–Zr alloys. J. Mater. Res. Technol..

[10] JaeJung G., Cho Y.-H., Kim S.-D., Kim S.-B., Lee S.-H., Song K., Euh K., Lee J.-M. (2020). Mechanism of ultrasound-induced microstructure modification in Al–Zr alloys. Acta Mater..

[11] Mohammadi A., Enikeev N.A., Murashkin M.Y., Arita M., Edalati K. (2021). Developing age-hardenable Al-Zr alloy by ultra-severe plastic deformation: Significance of supersaturation, segregation and precipitation on hardening and electrical conductivity. Acta Mater..

[12] Pozdniakov A., Barkov R., Prosviryakov A., Churyumov A., Golovin I., Zolotorevskiy V. (2018). Effect of Zr on the microstructure, recrystallization behavior, mechanical properties and electrical conductivity of the novel Al-Er-Y alloy. J. Alloys Compd..

[13] Zhang Y., Gu J., Tian Y., Gao H., Wang J., Sun B. (2014). Microstructural evolution and mechanical property of Al–Zr and Al–Zr–Y alloys. Mater. Sci. Eng. A.

[14] Knipling K.E., Dunand D.C., Seidman D.N. (2008). Precipitation evolution in Al–Zr and Al–Zr–Ti alloys during aging at 450–600 °C. Acta Mater..

[15] Saunders N., Rivlin V.G. (1986). Thermodynamic characterization of Al–Cr, Al–Zr, and Al–Cr–Zr alloy systems. Mater. Sci. Technol..

[16] Pauzon C., Buttard M., Després A., Chehab B., Blandin J.-J., Martin G. (2022). A novel laser powder bed fusion Al-Fe-Zr alloy for superior strength-conductivity trade-off. Scr. Mater..

[17] Zhang J., Hu T., Yi D., Wang H., Wang B. (2019). Double-shell structure of Al_3_ (Zr, Sc) precipitate induced by thermomechanical treatment of Al–Zr–Sc alloy cable. J. Rare Earths.

[18] Wu S.H., Xue H., Yang C., Cheng P.M., Zhang P., Kuang J., Zhang J.Y., Liu G., Sun J. (2021). Effect of Si addition on the precipitation and mechanical/electrical properties of dilute Al–Zr-Sc alloys. Mater. Sci. Eng. A.

[19] Glerum J.A., Kenel C., Sun T., Dunand D.C. (2020). Synthesis of precipitation-strengthened Al-Sc, Al-Zr and Al-Sc-Zr alloys via selective laser melting of elemental powder blends. Addit. Manuf..

[20] Wen S.P., Gao K.Y., Li Y., Huang H., Nie Z.R. (2011). Synergetic effect of Er and Zr on the precipitation hardening of Al–Er–Zr alloy. Scr. Mater..

[21] Fang Z.B., Wang R.M., Li C.Y., Miao C.H., Teng Y., Chen G.H., Tang W.M. (2022). Effect of aging on precipitation of second phase and properties of high-conductivity heat-resistant aluminum alloy wire. Equip. Environ. Eng..

[22] Zhang E.Q., Wen S.P., Hou J., Nie Z.R. (2018). Effect of annealing on microstructure and properties of cold-rolled Al-0.04Er and Al-0.08Zr alloys. Heat Treat. Met..

[23] Rakhmonov J., Poplawsky J., Allard L., Burns J., Qi J., Coello I., Dunand D.C., Bahl S., Perrin A., Haynes J.A. (2026). Additively-manufactured Al-0.3Zr-0.2Ce-0.2Cu alloy with high creep resistance and electrical conductivity. Acta Mater..

[24] Xu J., Wen S., Liu T.-H., Wang W., Nie N.-R. (2018). High-temperature coarsening behavior of precipitated phases in Al-Er-Zr/Hf alloys. Trans. Mater. Heat Treat..

[25] Li Y., Wen S.P., Gao K.Y., Huang H., Nie Z.R. (2013). Aging precipitation process of Al-Er-Zr alloy. Chin. J. Nonferrous Met..

[26] Yuan X., Wen S., Hou J., Wu X., Bolong L., Gao K., Huang H., Nie Z. (2020). Hot Deformation Behavior and Dynamic Precipitation in Al-Er-Zr Alloy. Rare Met. Mater. Eng..

[27] Wen S.P., Gao K.Y., Huang H., Wang W., Nie Z.R. (2013). Precipitation evolution in Al–Er–Zr alloys during aging at elevated temperature. J. Alloys Compd..

[28] Huang H., Wen S.P., Gao K.Y., Wang W. (2013). Age hardening behavior and corresponding microstructure of dilute Al-Er-Zr alloys. Metall. Mater. Trans. A.

[29] Wen S.P., Li B.L., Rong L., Gao K.Y., Huang H., Wang W., Nie Z.R. (2014). Strain induced rapid precipitation in Al-Er-Zr alloy. Mater. Sci. Forum.

[30] Liu T.H., Wen S.P., Wu X.L., Gao K.Y., Huang H., Wang W., Nie Z.-R. (2017). Combined Effect of Cold-Rolling and Aging on Precipitation and Recrystallization Behavior of Al-Er-Zr Alloy. Mater. Sci. Forum.

[31] Liu T.H., Wen S.P., Wu X.L., Huang H., Gao K.Y. (2017). Effect of trace element Hf on the precipitation process and recrystallization resistance of Al-Er-Zr alloys. Mater. Sci. Forum.

[32] Wang C., Qu C., Chen J., Li B., Liu K., Han Q. (2022). Performance of aging-treated heat-resistant Al–Cu–Sc wires. Mater. Lett..

[33] Orlova T., Mavlyutov A., Latynina T., Ubyivovk E., Murashkin M., Schneider R., Gerthsen D., Valiev R. (2018). Influence of severe plastic deformation on microstructure, strength and electrical conductivity of aged Al–0.4 Zr (wt.%) alloy. Rev. Adv. Mater. Sci..

[34] Belov N., Alabin A., Matveeva I., Eskin D. (2015). Effect of Zr additions and annealing temperature on electrical conductivity and microhardness of hot rolled Al sheets. Trans. Nonferrous Met. Soc. China.

[35] Chao R., Guan X., Guan R., Tie D., Lian C., Wang X., Zhang J. (2014). Effect of Zr and Sc on mechanical properties and electrical conductivities of Al wires. Trans. Nonferrous Met. Soc. China.

[36] Jiang S.Y., Wang R.H. (2018). Manipulating nanostructure to simultaneously improve the electrical conductivity and strength in microalloyed Al-Zr conductors. Sci. Rep..

[37] Zhang Y., Zhou W., Gao H., Han Y., Wang K., Wang J., Sun B., Gu S., You W. (2013). Precipitation evolution of Al–Zr–Yb alloys during isochronal aging. Scr. Mater..

[38] Belov N.A., Korotkova N.O., Akopyan T.K., Timofeev V.N. (2021). Structure and Properties of Al-0.6% Zr-0.4% Fe-0.4% Si (wt.%) Wire alloy manufactured by electromagnetic casting. JOM.

[39] Guan R., Shen Y., Zhao Z., Wang X. (2017). A high-strength, ductile Al-0.35 Sc-0.2 Zr alloy with good electrical conductivity strengthened by coherent nanosized-precipitates. J. Mater. Sci. Technol..

[40] Liu L., Jiang J.-T., Zhang B., Shao W.-Z., Zhen L. (2019). Enhancement of strength and electrical conductivity for a dilute Al-Sc-Zr alloy via heat treatments and cold drawing. J. Mater. Sci. Technol..

[41] Knipling K.E., Karnesky R.A., Lee C.P., Dunand D.C., Seidman D.N. (2010). Precipitation evolution in Al–0.1 Sc, Al–0.1 Zr and Al–0.1 Sc–0.1 Zr (at.%) alloys during isochronal aging. Acta Mater..

[42] Booth-Morrison C., Dunand D.C., Seidman D.N. (2011). Coarsening resistance at 400 C of precipitation-strengthened Al–Zr–Sc–Er alloys. Acta Mater..

[43] Kang W., Li H.Y., Zhao S.X., Han Y., Yang C.L., Ma G. (2017). Effects of homogenization treatments on the microstructure evolution, microhardness and electrical conductivity of dilute Al-Sc-Zr-Er alloys. J. Alloys Compd..

[44] Zhou W.W., Cai B., Li W.J., Liu Z.X., Yang S. (2012). Heat-resistant Al–0.2 Sc–0.04 Zr electrical conductor. Mater. Sci. Eng. A.

